# How much of the labor in African agriculture is provided by women?^☆^

**DOI:** 10.1016/j.foodpol.2016.09.017

**Published:** 2017-02

**Authors:** Amparo Palacios-Lopez, Luc Christiaensen, Talip Kilic

**Affiliations:** a Living Standards Measurement Study (LSMS), Survey Unit, Development Data Group, The World Bank, United States; b Jobs Group, The World Bank, United States; c Living Standards Measurement Study (LSMS), Survey Unit, Development Data Group, The World Bank, Italy

**Keywords:** Gender, Labor, Agriculture, Sub-Saharan Africa

## Abstract

The contribution of women to labor in African agriculture is regularly quoted in the range of 60–80%. Using individual, plot-level labor input data from nationally representative household surveys across six Sub-Saharan African countries, this study estimates the average female labor share in crop production at 40%. It is slightly above 50% in Malawi, Tanzania, and Uganda, and substantially lower in Nigeria (37%), Ethiopia (29%), and Niger (24%). There are no systematic differences across crops and activities, but female labor shares tend to be higher in households where women own a larger share of the land and when they are more educated. Controlling for the gender and knowledge profile of the respondents does not meaningfully change the predicted female labor shares. The findings question prevailing assertions regarding substantial gains in aggregate crop output as a result of increasing female agricultural productivity.

## 1 Introduction

Women are commonly considered to perform the bulk of work in African agriculture. Combined with new evidence of a non-negligible gender gap in agricultural productivity, this has motivated increased attention to raising agricultural productivity among African women.^1^ Doing so is not only seen as important for empowering Africa’s women and improving the development outcomes of the next generation, but also as an important vehicle to increase Africa’s food supply, a key objective on the agenda of African and international policymakers (AGRA, 2012).^2^

This paper revisits the first premise of this reasoning, i.e. that women perform the bulk of work in African agriculture. Systematic data on women’s labor contribution to agriculture are hard to come by. As such, it is no surprise that the widely shared notion that women in Sub-Saharan Africa (SSA) are responsible for 60–80% of the agricultural labor supplied, traces back to an undocumented, 1972 quote in a more general study of women’s contribution to development.^3^ The statistical basis for these numbers has been questioned before (Jackson, 2005, Doss, 2014, Doss et al., 2011).

Taking the female share of the agricultural labor force as a proxy (calculated as the total number of women economically active in agriculture divided by the total population economically active in agriculture), FAO (2011) suggests that women’s labor contribution to African agriculture is slightly less than half. Using more reliable, but non-nationally representative case study evidence from time use surveys, estimates reported in the same study range from 30% time contribution by women to agricultural activities in The Gambia, to 60–80% in different parts of Cameroon. In addition to the wide variation across countries (and at times within countries), the time use surveys reveal important differences in time allocation across crops, agricultural activities and technology. FAO (2011) concludes with a call for more systematic evidence on women’s labor contribution to agricultural production in SSA.

The Living Standards Measurement Study – Integrated Surveys on Agriculture (LSMS-ISA) initiative provides a unique opportunity to start filling this void.^4^ Under the LSMS-ISA initiative, nationally-representative household surveys were fielded during the 2009–2011 period (and at least once thereafter) in six African countries (Ethiopia, Malawi, Niger, Nigeria, Tanzania and Uganda). Together, these countries cover a wide array of agro-ecological zones and farming systems, and make up approximately 40% of the region’s population. Detailed labor input data was collected at the plot-level for each household member and across activity domains, enabling systematic estimation of women’s time contribution to agricultural (crop) production as well as a systematic comparison across settings, crops and activities.

The primary objective of this paper is to provide detailed, systematic and nationally representative evidence on female labor input into agricultural activities for a series of countries in SSA. By putting the premise of the reasoning advanced above on more solid empirical footing, it helps assess its validity, while informing the policy dialogue on gender and agriculture more broadly. The focus is on time allocation to crop production. Crop production continues to make up the bulk of agricultural GDP in most African countries. Gender disaggregated data on labor input in livestock activities are also not yet systematically available. Food processing, which is typically the exclusive domain of women, is further excluded, consistent with the time use surveys reviewed by FAO (2011) and the aforementioned claims regarding female labor share in agriculture. The study further probes into the underlying processes and factors that affect, at the household-level, women’s time allocation to crop production. The robustness of the findings is assessed by controlling for possible gender and knowledge bias in reporting, which may occur when responses on labor input come from proxies (i.e. other household members) as opposed to self-reporting.^5^

By way of headline number, the population-weighted female share of labor in crop production across the six African countries examined here is 40%. This is substantially less than the widely quoted figures of 60–80%. Consistent with FAO (2011), wide variation is recorded across (and within) countries, with the country-level shares ranging from 56% in Uganda to only 24% in Niger. For Nigeria as a whole, the share is 37%; it is only 32% in the North, and 51% in the South.^6^ The empirical finding that women do not disproportionately contribute to crop production proves robust to possible gender and knowledge biases in reporting. The primary factor underlying differences in female labor input across households is the gender composition of the household. There is little systematic difference across countries in female labor provision across crops or agricultural activities. These findings attenuate the premise on which recent calls for boosting agricultural output by increasing female agricultural (whether land or labor) productivity are based. However, they do in and of themselves neither invalidate nor validate the conclusion of the argument. Other premises, such as substantial gender gaps in land productivity, may support the same conclusion. Additionally, there may be many other reasons for fostering female agricultural productivity, beyond boosting agricultural output, such as female empowerment. The key objective here is to put the policy dialogue on solid empirical footing.

The paper proceeds by describing the data in more detail and discussing the key methodological considerations in Section 2. The empirical findings regarding the female labor share in crop production in Africa, their robustness, and the key correlates are presented in Section 3. Section 4 concludes.

## 2 Data and methods

### 2.1 Understanding the information base

Nationally-representative time use surveys or labor force surveys that depict the relative labor contributions of men and women in agriculture within an appropriate reference period remain largely lacking (FAO, 2011). As such, the nationally-representative household surveys conducted under the LSMS-ISA initiative present a rather unique opportunity to study and compare the female labor share in agriculture across diverse settings. These data form the information base of the paper. In particular, the analysis uses the data from Ethiopia Socioeconomic Survey 2011/12, Malawi Third Integrated Household Survey 2010/11, Niger l’Enquête Nationale sur les Conditions de Vie des Ménages et l’Agriculture 2011, Nigeria General Household Survey – Panel 2012/13, Tanzania National Panel Survey 2010/11, and Uganda National Panel Survey 2010/11.^7^

In each LSMS-ISA country, all sample households are administered a multi-topic Household Questionnaire.^8^ Agricultural households, who are defined as owning and/or cultivating land and/or owning livestock, are additionally administered an Agricultural Questionnaire. The latter records (i) geo-referenced plot locations and Global Positioning System (GPS)-based plot areas, (ii) collects plot-level information on input use, cultivation and production, (iii) identifies the household members that manage and/or own each plot, and (iv) most importantly, solicits individual-disaggregated labor input at the plot-level. The information is collected separately for each agricultural season in the country (if there is more than one), and often in two visits, with information on the post-planting/pre-harvest and the post-harvest outcomes collected during the first and second visit, respectively.

While the data are collected at the plot-level in all countries, the quantification of the labor input at the plot level differs slightly across countries. The surveys in Malawi, Nigeria and Ethiopia collect data on the number of weeks of work provided by each household member on each plot, differentiated by activity (land preparation and planting, weeding/fertilizing/other non-harvest activities, and harvesting). This is complemented by further probing regarding the average number of days per week and the average number of hours per day worked on each plot, separately for each activity. The surveys in Tanzania and Niger solicit the number of days worked by each member of the household on each plot, separately for each activity domain. Finally, in Uganda, the total number of days worked on a plot (by all household members and across all activities) are collected first. Subsequently, the household members who worked on the plot are identified. In the analysis, the total number of days worked are distributed equally among all household members working on the plot.^9^

Only labor input of adult household members (above 15 years old) is considered in the results reported below. They contribute almost all labor for crop production.^10^ To obtain country-level estimates of the female labor share in crop production, the plot-level unit record data of each household member’s labor input are cleaned and compiled as follows. First, the individual-disaggregated data on labor inputs collected at the plot-level for various activity domains are compiled into a household member-level database. Second, outliers in individual labor aggregates across plots and activities (in hours or days, depending on the survey) are identified based on common sense parameters and are replaced by missing values. For each individual, the number of weeks for land preparation and planting, for weeding, fertilizing and other non-harvest activities, and for harvesting are capped at 13, 26, and 13 weeks, respectively. A typical week was taken to be no more than 7 days, and the number of hours in a typical work day was capped at 12 h. Third, the top and bottom 1% of individual-level labor aggregate values, within each region, sex and age combination, are further tagged as outliers and replaced by missing values. Finally, following Scheffer (2002), the resulting missing values^11^ were imputed using single regression-based imputation.^12^

Application of these procedures yields the information base for estimating the female share of labor in crop production and its correlates. To obtain the national female labor share, all agricultural households are considered (both rural and urban). Estimates are weighted using the sampling weights and in accordance with the complex survey design (i.e. stratification and clustering) in each country, aggregated by each gender and then divided by the total crop labor time spent by adult household members in the country (or in a given category, when disaggregating within country across categories).

### 2.2 Detecting possible reporting bias

As Bardasi et al. (2011) discuss, both the level of detail on employment questions and the type of respondent (proxy versus self) may affect labor statistics and their comparability across countries. Here, the focus is not on the *levels* of engagement, but rather on the relative engagement by men and women, i.e. the agricultural labor *shares*. To the extent that potential over- or underreporting due to survey design issues affect male and female household members similarly (and in the same direction), survey design issues (including the use of recall data) should be less of a concern when estimating shares, though potential bias cannot be excluded.

Concerning the potential effect of the detail of the questionnaire, Bardasi et al. (2011) find a decline in male labor participation in agriculture and a (statistically non-significant) increase in female labor participation when a short as opposed to a detailed labor force participation module is used. The use of short modules would thus lead to an underestimation of the female labor share. Here, very detailed labor modules are used, with questions about the amount of time spent on each plot by each member per activity domain. This greatly reduces the potential risk of bias (such as overstating male and understating female agricultural labor participation), which might occur if there were a lack of probing about labor engagement in agriculture across household members.

The assumption of gender neutrality in *level* reporting is likely also somewhat tenuous when it comes to self-versus proxy reporting.^13^ Men may systematically over- or underreport the labor contribution of women, and vice versa. As Bardasi et al. show, however, the direction of potential bias is not clear a priori. The authors find that response by proxy has no effect on female labor statistics (compared with self-reports), but that it yields substantially lower male employment rates, mostly due to underreporting of the agricultural activity. They further find that the large impacts of proxy responses on male employment rates are attenuated when proxy informants are spouses or individuals with some schooling.

This highlights a second important feature of the respondent (in addition to their gender), i.e. their familiarity with each member’s agricultural labor input. In the surveys supported under the LSMS-ISA initiative, when possible, plot-level modules are administered to the manager of the plot, who is arguably the most knowledgeable individual about the household labor input on the plot.

To assess the robustness of the findings, the sensitivity of the estimates of the female labor share in crop production to the gender and knowledge profile of the respondent is assessed. The core specification for this purpose is:

(1) $$L_{\mathrm{fi}}=\alpha +R_{f}+R_{k}+\beta X_{i}+\gamma D_{i}+\varepsilon_{i}$$

where $L_{\mathrm{fi}}$ is the female labor share in crop production in household *i*, $R_{f}$ is a dummy variable that is equal to 1 if the respondent is female for the majority of the household plots,^14^ $R_{k}$ is a dummy variable which is equal to 1 if the respondent is knowledgeable about the labor input (respondents are considered knowledgeable if their plot labor share averaged across plots was more than 50%),^15^ $X_{i}$ is a vector of other household-level covariates affecting the outcome of interest, $D_{i}$are location fixed effects, and $\varepsilon_{i}$ is the stochastic error term, randomly distributed across households. Eq. (1) is estimated by country using OLS.^16^

Inclusion of the household level covariates *X* further helps control against potential selection bias of the respondent. For example, it is important to control for the demographic composition of the household’s labor pool, i.e. the age and gender composition of all adults and in particular whether the household is female headed without male adults. Failure to control for the latter could significantly upwardly bias the effect of the gender of the respondent on the estimated female labor share. Women in such households are the only adult labor providers as well as the respondent.^17^ Other controls relate to (i) proxies for the availability of household labor substitutes, (ii) proxies for culture-specific gender roles that determine the capacity of men and women to allocate labor time across reproductive (household) and productive (economic) activities, and (iii) socio-economic factors that may explain why women could work more or less than men in agriculture (such as proxies for employment opportunities off the farm). The reasons for their inclusion and the indicators used are discussed in more detail below.

Estimation of Eq. (1) helps assess whether the gender and knowledge of the respondent, ceteris paribus, affect the reported female labor share (i.e. due to survey design) and by how much. It also helps put bounds on the nationally estimated female labor shares. This can be done by comparing the predicted female share for the sample as a whole, with the predicted female share assuming all respondents are knowledgeable and either female (if one believes females to provide a more correct estimate of both women’s and men’s labor contribution) or male (if one believes males to provide a more correct estimate of both women’s and men’s labor contribution). The degree of deviation from the sample estimate will depend on (i) the share of knowledgeable and female/male respondents in the original sample, and (ii) the size of the effect of the gender and knowledge of the respondent. The gender and knowledge “bias” could also be examined separately. To see this clearly, note that there are essentially four scenarios with potentially differential female agricultural crop labor share estimates. Following the set up in Eq. (1) and denoting the estimated coefficients and shares by “^” yields four scenarios:

Scenario 1 All respondents are knowledgeable and female, and women respond most truthfully on both women’s and men’s labor contributions (i.e. no knowledge or male respondent bias):

(2) L1fi^=[α^+β^Xi+γ^Di]+R^f+R^k

Scenario 2 All respondents are knowledgeable and male, and men respond most truthfully on both women’s and men’s labor contributions (i.e. no knowledge or female respondent bias):

(3) L2fi^=[α^+β^Xi+γ^Di]+R^k

Scenario 3 None of the respondents are knowledgeable, all are female, and women respond most truthfully on both women’s and men’s labor contributions (i.e. knowledge bias but no male respondent bias):

(4) L3fi^=[α^+β^Xi+γ^Di]+R^f

Scenario 4 None of the respondents are knowledgeable, all are male, and men respond most truthfully on both women’s and men’s labor contributions (i.e. knowledge bias but no female respondent bias):

(5) L4fi^=[α^+β^Xi+γ^Di]

By comparing the predicted female labor share estimates from Eqs. (2), (3), (4), (5) to the predicted female labor share, Lfi^, from Eq. (1), the effect of the potential gender and knowledge bias tied to the respondent can be assessed.

### 2.3 Identifying correlates of the female share of agricultural labor

The covariates included as part of vector *X* in Eq. (1) also shed light on the different factors that underlie labor allocation to agriculture across gender lines. This has been under-researched thus far and the literature provides only limited, and mainly indirect, guidance regarding the core factors to be considered (Blackden and Wodon, 2006). The results below provide some exploratory guidance, and should be considered as such. No causality in estimation or interpretation is purported. The multivariate analysis is applied in three countries (Malawi, Niger and Nigeria) that exhibit different degrees of female contribution to crop production.

The following correlates have been retained for exploration, grouped under three broad headings: (i) the availability of household labor and substitutes; (ii) the culture-specific gender roles that determine the capacity of men and women to allocate labor time across reproductive (household) and productive (economic) activities, (iii) socio-economic factors that may explain why women could work more or less than men in agriculture. At the household-level, the number and gender composition of children, adult, and elderly (as well as the marital status – polygamous or not) affect individuals’ time use patterns within and across sectors in general, and women’s labor input into agriculture in particular (Nankhuni, 2004, Blackden and Wodon, 2006).

In addition, hired and exchange labor may substitute for household labor and they may do so differently for men and women. This might, for example, be the case if labor is mainly hired for plowing, which is often considered a male activity, or if community labor exchange programs are confined to males only. Furthermore, missing or incomplete labor markets (Dillon and Barrett, 2014, Palacios-Lopez and Lopez, 2015) may affect female-managed plots more than male-managed plots. Similarly, if the substitution of capital for labor is gender-sensitive, the use of labor-saving technologies, such as agricultural implements, might affect the level of women’s labor input into agriculture. In addition to the number of adult household members by gender/age groups, the total number of hired and exchange labor days as well as the number of agricultural implements owned and accessed are included.

Second, culture-specific gender roles determine the capacity of men and women to allocate labor time across economically productive activities and to respond to economic incentives (Ilahi, 2000, Blackden and Wodon, 2006). Domestic responsibilities, such as childcare and caring for the sick, water and firewood collection, and cooking, are usually in the female domain. They are easier to combine with on-farm work close to the homestead than with off-farm employment (Blackden and Morris-Hughes, 1993, Palacios-Lopez and Lopez, 2015). To capture the potential effects of these dynamics on the female agricultural labor share, the number of children in the household under 5 and between 6 and 14 years old are included as well as the percent of female and male adults suffering from chronic diseases. Given the possibility of differential control over the proceeds from cash and food crops by gender, the overall female labor share in crop agriculture may further depend on the land allocation to different crops (as well as the overall amount of land cultivated).^18^ As these differential gender controls are deeply culturally determined, with potentially substantial differences across ethnic groups, the ethnicity of household head is further controlled for.

Socio-economic factors behind individual labor allocation decisions across sectors include gender differences in education, which may affect the scope of off-farm opportunities available to women compared to men. At the same time, gender discrimination in access to and returns from off-farm employment (Doss et al., 2011, Hertz et al., 2009), which may partially be a product of culture, could lead rural women (including the more educated) to still prefer work on the farm. Household economic status may further influence individual labor allocation decisions. Poorer households may need all of their members to work, while richer households may have better access to off-farm opportunities, which men may also be more likely to take up. To control for differences in off-farm employment opportunities the travel time to the nearest population center of 20,000 is included. To see how livestock ownership affects agricultural labor allocation across gender, the number of tropical livestock units (TLU) is also included. Finally, the larger is the share of land women own, the larger is their expected share of a household’s agricultural labor time, ceteris paribus.

## 3 Results

### 3.1 Female share of agricultural labor is 40% on average, but varies across countries

**Fig. 1:**
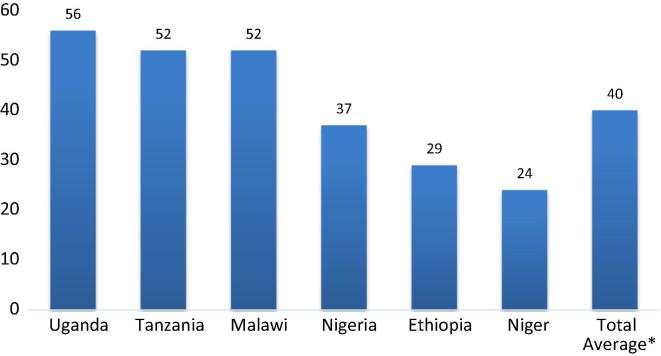
Female share of agricultural labor (%) by country.

The population-weighted average female share of labor in crop production across the six countries examined is 40% (Fig. 1). This is substantially less than in the much cited 1972 quote which holds that “Few persons would argue against the estimate that women are responsible for 60–80 [percent] of the agricultural labor supplied on the continent of Africa.” It is also somewhat lower than FAO’s (2011) estimate of about 50%, based on agricultural employment categories only (as opposed to time use).^19^ To be sure, even though the six countries represent 40% of SSA’s population and cover a wide variety of settings, they are not statistically representative for the continent either. At the same time, this overall headline number provides a useful antidote.

More important than the overall headline number is the variation across the countries. At 56%, the estimated female share of agricultural labor is highest in Uganda, followed by Tanzania (52%) and Malawi (52%). Taking the female share in the total population as a natural benchmark, these are also three countries where the female share in the population is slightly above half (52, 53 and 51% respectively (Table 1, column 2). In contrast, women contribute less than a quarter of the overall amount of labor to crop production in Niger (24%) and only slightly more in Ethiopia (29%).^20^

The findings for Nigeria are especially illuminating. On average about 37% of labor in crop production is contributed by women. Yet, this reduces to less than a third (32%) when looking at northern Nigeria only. In southern Nigeria, the share is similar to the shares found in eastern and southern Africa (51%) (Table 1, column 1). This tallies well with expectations. The ability of the data to distinguish these differences within Nigeria provides confidence in the approach. It also underscores the heterogeneity in women’s time allocation in agriculture, even within countries.

Very similar results are obtained in all countries when using the data without application of the cleaning and/or imputation procedures, suggesting that the outliers and missingness tend to affect responses on both male and female agricultural labor inputs similarly.^21^ By way of comparison, Table 1 (column 3) also reports the female agricultural labor shares as estimated from the main employment activity of all persons of working age from the ILO database. The results are very close to the LSMS-ISA based estimates (Table 1, column 1). The ILO estimates comprise all agricultural activities (crop and animal production, forestry and logging, fishery and hunting); they are based on the International Standard Industrial Classification. The omission of the labor allocation to livestock and other agricultural subsectors beyond crop production in the LSMS-ISA estimates does not seem to affect the overall findings. Nonetheless, to be conclusive further data collection and analysis is needed, especially in countries where livestock is important.^22^

Table 2, Table 3 explore potential heterogeneity in labor allocation by gender across aggregate crop categories^23^ and crop production activities. For instance, men are often thought to allocate disproportionately more of their time to (non-edible) cash crops, while women are believed to concentrate more on the production of staple and other food crops. Compared to the overall female share of agricultural labor, the female share allocated to non-edible crops is slightly lower in Malawi and Uganda, while it is slightly higher in Tanzania and a lot higher in southern Nigeria (Table 2).^24^ Overall, while there is some variation in the female share of agricultural labor across crop categories within countries (especially Niger, Nigeria), these patterns are not generalizable across countries.

Turning to the crop production activities, land preparation is often considered a “male activity.” There are some signs of this, though only in Ethiopia and Niger (Table 3). There is hardly any variation in female labor allocation across agricultural activities in Tanzania, Malawi and Nigeria.^25^ This is likely linked to the low degree of mechanized land preparation in SSA, with the exception of Ethiopia (and to some extent also in Niger), where the use of draught animals is more widespread (Sheahan and Barrett, 2014).^26^

Furthermore, there is an emerging debate about the declining interest of African youth in agriculture (Bezu and Holden, 2014, Maiga et al., 2015). To explore whether this affects young male and female adults differently, Table 4 presents female labor shares in agriculture by age group. There is no systematic difference in labor shares for the 15–30 or for the 30–45 year olds across countries, with the exception of Nigeria, where women among 30–45 year olds carry a substantially larger share of the labor input. This suggests that there is no real gender difference in the labor contribution of youth to agriculture, beyond Nigeria. Regarding the other age groups, girls under 15 years old tend to participate slightly less in crop production than boys, possibly because other related household tasks, except in Ethiopia where they take on a larger share. Lastly, women above 60 years old participate less in crop related activities.

### 3.2 Controlling for respondent characteristics does not fundamentally change the core insight

The coefficients from the OLS regressions of household-level female labor share in crop production are in Table 5. They have been estimated for three countries (Malawi, Nigeria, Niger), which cover the spectrum of female agricultural labor shares observed across the LSMS-ISA countries (52, 37 and 24%, respectively). The regressions were also run for North and South Nigeria separately. In Malawi and Nigeria, the gender of the respondent was also recorded. In Malawi, slightly more than half of the respondents were female (54%) (Appendix Table B1) and slightly more than half of the respondents contributed more than 50% of the labor on all the plots they reported on. The corresponding numbers are 84 and 55% in Nigeria.

Controlling for a host of demographic, cultural and socio-economic characteristics, the reported female labor share in Malawi is estimated to be four percentage points higher when the respondent is knowledgeable and six percentage points higher when the respondent is female. In Nigeria, the opposite is observed. More knowledgeable respondents tend to report a lower female share of labor, as do female respondents (though the latter effect is not statistically significant). This suggests that there can be a lingering effect of the characteristics of the respondent on the reported labor shares, after controlling for a host of factors. The direction of these effects, however, can go either way.

One way to gauge the possible effect is to establish a range by predicting the estimated female labor shares for the extreme cases when all respondents are knowledgeable and female as well as the case when all respondents are not knowledgeable and male (cases 2 and 5 in Table 6). Doing so, situates the female agricultural labor share between 60 and 50% and between 24 and 38% in Malawi and Nigeria respectively, compared with a sample predicted share of 56 and 32% respectively. Put differently, the point estimates may be four to eight percentage points higher or lower when taking these extreme cases.^27^ Clearly, more work is needed to more accurately establish the role of the characteristics of the respondent in estimating the female labor share. Even so, the key point advanced here, that the average female agricultural labor share across both countries is well below the shares commonly quoted in policy circles, stands.

### 3.3 Gender composition of the household’s labor force, mechanization, female education and land ownership emerge as consistent correlates

As expected, the female labor share in crop production is strongly associated with the gender composition of the household’s labor force (Table 5). Similarly, adult women in female headed households devote a much larger share of their labor time to crop production, with the effect most pronounced when there are no males in the household. These results hold partly by design (especially the last one). As such they act as control variables. Female labor participation in agriculture is also higher in polygamous households in Malawi and Niger (though not in Nigeria).

There are signs of gender differentiated labor substitution through machinery, which reduces the female labor share.^28^ In contrast, household’s female labor share tends to increase when labor is brought in from outside the household (through hiring or labor exchange programs). Yet this holds only in some countries and when present, the gender differentiated effects of all household labor substitution mechanisms are small in magnitude (coefficients of less than 0.00).

There are also indications that culturally defined roles affect the female labor share in crop production, albeit differently across countries. In Malawi, women tend to contribute a larger share of the household’s crop production time in households with more children, and in Nigeria, they contribute a larger share when the household houses a chronically ill adult male. Consistent with the bivariate findings discussed above, the findings on the share of land devoted to different crops suggests that there is slightly more female labor involvement in the production of cereals and legumes in Malawi compared with their involvement in non-edible crops, while the reverse holds for Niger. Given the small share of land devoted to non-edible crops in Niger, the latter results should be taken with caution. No significant difference across crops was found in Nigeria.

Turning to the economic factors, more educated women tend to provide a larger share of the household’s labor input into crop production. Women also contribute a larger share if they own the land. Both effects hold across countries, while the effect of household wealth differs by country (increasing the female share in Malawi, and reducing it in Nigeria and Niger). Access to earned income from off-farm activities (captured by a dummy variables which is one if the household has earned income from off-farm activities) only reduces the female labor share in crop production in Nigeria (and increases it in North Nigeria when it concerns unearned non-farm income). This may point to specialization across activities along gender lines in more developed economies. There is no effect in the other countries and there are no statistically significant associations between the outcome of interest and the proxies for market access.

The results so far have only considered the female labor share in crop production. One way to explore the effects of livestock on women’s agricultural labor contribution is to see whether their labor share in crop production is affected by livestock ownership. No effect was found in Malawi or Niger, but there was a statistically significant negative effect in (Northern) Nigeria. This may point to some substitution effects, though given that the average TLU is only 0.03, it would not meaningfully affect the estimated female labor share in crop (or even overall agricultural) production.

## 4 Conclusion

Using recent, activity-specific plot level information on individual labor input into crop production from six Sub-Saharan African countries, this study has revisited the premise that women provide 60–80% of Africa’s agricultural labor. Average labor contribution to crop production in these six countries is estimated at 40% instead, though differences exist across countries. The female labor share amounts to slightly more than 50% in Uganda, Tanzania and Malawi (56, 52 and 52%, respectively), which is also consistent with the slightly higher share of women in these populations (52, 53, and 51%, respectively). It is well below half in Nigeria, Ethiopia and Niger, where the female labor shares are estimated at 37, 29 and 24%, respectively. Within Nigeria, the shares differ starkly between the north and the south, 32% in northern Nigeria and 51% in southern Nigeria, consistent with expectations. While the gender and knowledge profile of the respondent affect these estimates somewhat, there is no uniform pattern either way across countries and controlling for the effects of the respondent characteristics (resulting in four to eight percentage points over- or underestimation) does not overturn the key conclusion that female labor shares in African crop agriculture tend to be well below 60–80%.

Cross-country generalizable patterns in the factors affecting women’s contributions to labor in agriculture were few and far between. Women tended, for example, to be slightly less involved in cash crop production in some countries (Malawi, Uganda) but not in others (Tanzania, southern Nigeria). Similarly, there was no clear difference in female labor shares across agricultural activities (land preparation, planting/weeding, harvesting), except in Ethiopia (and to a lesser extent in Niger), where women were relatively less involved in land preparation. Animal traction is also much more common in these countries, while Africa’s agricultural mechanization remains limited in general. Among the few consistent patterns across countries are an observed increase in female labor shares when women’s share in the household labor force increases (partly by design), when they are more educated and when they own the land. There are also incipient signs that women’s labor share in crop production decreases with mechanization.

The implications for policy are twofold. First, the lower than commonly-referenced female labor shares, which are well below 50% in some countries, do not, as such, support (universally) disproportionate focus on female farmers to boost crop production. That said, could concerted policy attention to women to boost agricultural output in Africa still be argued for based on the gender gap in agricultural productivity? Caution is counseled here, given the importance of using consistent metrics in analyzing the costs and benefits from different interventions. The estimated gender gaps in agricultural productivity are not based on differences in returns to male and female labor time spent on crop production within the household (the metric revisited in this paper). They are calculated based on differences in land productivity between male- and female-managed plots.^29^ With female-managed plots on average less than 25% of the plot population,^30^ full elimination of the gender gap in land productivity (estimated at 25% at most) would increase aggregate crop output by no more than 6.25% (and often less).^31^

Second, to be sure, all this should not be taken to mean that investment in raising female labor productivity in agriculture cannot be a high return activity to reach other objectives, including female empowerment and improved nutritional outcomes of children, especially when compared to other options. Establishing this requires further research for which nationally representative and gender-disaggregated household survey data on time use and intra-household control of income and productive resources will be key. The new survey rounds supported under the LSMS-ISA initiative are making useful steps in this direction, creating promising opportunities for future research on gender and agriculture in Sub-Saharan Africa.

## Figures and Tables

**Table A1:** LSMS-ISA surveys informing the analysis – key features & approaches to household agricultural labor data collection.

Country/year	Name of survey	Agency	Sample size/coverage	Field work	Activities	Unit reported	How it was asked
Ethiopia 2011/2012	Socio-Economic Survey (SES)	Ethiopia Central Statistics Agency (CSA)	4000 households/Rural areas and small towns	September 2011 - February 2012	Land Preparation Planting, Weeding Harvesting	Hours per person working on each plot	Number of weeks, average number of days per week, average number of hours per day
Malawi 2010/2011	Third Integrated Household Survey (IHS3)	Malawi National Statistical Office	12,271 households/Nationally representative	March 2010 - April 2011	Land Preparation Planting, Weeding Harvesting	Hours per person working on each plot	Number of weeks, average number of days per week, average number of hours per day
Niger 2011	l’Enquête Nationale sur les Conditions de Vie des Ménages et l’Agriculture (ECVMA)	Niger National Institute of Statistics (NIS)	4000 households/Nationally Representative	July 2011 - December 2011	Land Preparation Planting, Weeding Harvesting	Days per person working on each plot	
Nigeria 2010/2011	General Household Survey (GHS) – Panel Component	Nigeria National Bureau of Statistics (NBS)	4716 households/Nationally Representative	September - November 2012 and February -April 2013	Land Preparation Planting, Weeding Harvesting	Hours per person working on each plot	Number of weeks, average number of days per week, average number of hours per day
Tanzania 2010/2011	Tanzania National Panel Survey (TZNPS)	Tanzania National Bureau of Statistics (NBS)	3924 households/Nationally Representative	October 2010 - September 2011	Land Preparation Planting, Weeding Harvesting	Days per person working on each plot	
Uganda 2010/2011	Uganda National Panel Survey (UNPS)	Uganda Bureau of Statistics (UBOS)	2716 households/Nationally Representative	October 2010 - September 2011	Total labor across all activities.	Total number of days per plot	Total number of days per plot, with follow up question on the household members who contributed labor to that plot. The total number of days were distributed evenly among the persons that worked on the plot

**Table B1:** Descriptive statistics for Malawi.

Variable	Mean	Std Err.	[95% Conf. Int.]		Min	Max
*Outcome variable*						
Household female share of agricultural labor	0.56	0.0	0.5	0.6	0.0	1.0

*Survey methodology*						
Respondent knows (works at least 50% on the plot)	0.53	0.0	0.51	0.54	0	1
Respondent Female (in at least 50% on the plots)	0.54	0.0	0.52	0.55	0	1
Female manager in majority of household plots, land weighted	0.29	0.0	0.3	0.3	0	1

*Demographic factors*						
*Gender & age composition of household labor pool*						
# of male HH members 15–39	0.80	0.0	0.8	0.8	0.0	7.0
# of Female HH members 15–39	0.88	0.0	0.9	0.9	0.0	5.0
# of Male HH members 40–59	0.24	0.0	0.2	0.3	0.0	2.0
# of Female HH members 40–59	0.25	0.0	0.2	0.3	0.0	2.0
# of Male HH members 60+	0.12	0.0	0.1	0.1	0.0	3.0
# of Female HH members 60+	0.15	0.0	0.1	0.2	0	3
Household head: female, with male†	0.10	0.0	0.1	0.1	0	1
Household head: female, with no male†	0.15	0.0	0.1	0.2	0	1
Household is polygamous†	0.08	0.3	0.1	0.1	0	1
Household size	4.74	0.0	4.7	4.8	1	17
*Household labor substitutes*						
Agricultural implements and machinery access	0.47	0.0	0.4	0.5	−3.3	9.7
Total hired labor (Days)	2.94	0.1	2.7	3.1	0	66
Total exchange labor (Days)	1.04	0.0	0.9	1.1	0	27

*Cultural roles*						
*Competing demands on time*						
# of HH members 0–5	0.97	0.0	0.9	1.0	0	7
# of HH members 6–14	1.32	0.0	1.3	1.4	0	9
% of female adult HH members suffering from chronic disease	0.08	0.0	0.1	0.1	0	1
% of male adult HH members suffering from chronic disease	0.05	0.0	0.0	0.1	0	1
*Household head ethnicity*						
Chewa†	0.55	0.0	0.5	0.6	0.0	1
Tumbuka†	0.10	0.0	0.1	0.1	0.0	1
Yao†	0.13	0.0	0.1	0.1	0.0	1
Nyanja†	0.05	0.0	0.0	0.1	0.0	1
Total land cultivated by the household	0.75	0.0	0.7	0.8	0.0	13.8
% of cultivated land under cereals	0.86	0.0	0.9	0.9	0.0	1
% of cultivated land under legumes	0.07	0.0	0.1	0.1	0.0	1
% of cultivated land under roots & tubers	0.01	0.0	0.0	0.0	0.0	1
% of cultivated land under fruits & vegetables	0.00	0.0	0.0	0.0	0.0	1

*Economic reasons*						
Maximum years of schooling among male HH members	5.49	0.0	5.4	5.6	0.0	17
Maximum years of schooling among female HH members	4.71	0.0	4.6	4.8	0.0	16
Wealth index	−0.91	0.0	−0.9	−0.9	−2.2	16
Livestock ownership (TLUs)	0.02	0.0	0.0	0.0	0.0	4
Access to non-farm earned income†	0.44	0.5	0.4	0.4	0.0	1
Access to non-farm non-earned income†	0.50	0.5	0.5	0.5	0.0	1
% of land owned by female HH members	0.40	0.0	0.4	0.4	0.0	1
Maize production potential (kg/HA, Low Input Maize)	1000	9	982	1018	1	2584
Travel time to nearest 20 K population center	0.94	0.0	0.9	1.0	0.0	5
Household: rural†	0.96	0.0	1.0	1.0	0.0	1

**Table B2:** Descriptive Statistics for Nigeria.

Variable	Mean	Std. Err.	[95% Conf. Interval]		Min	Max
*Outcome variable*						
Household female share of agricultural labor	0.32	0.01	0.30	0.33	0.00	1.00

*Survey methodology*						
Respondent knows (works at least 50% on the plot)	0.55	0.01	0.53	0.57	0.00	1.00
Respondent female	0.16	0.01	0.14	0.17	0.00	1.00
Female manager in majority of household plots, land weighted	0.15	0.01	0.14	0.17	0.00	1.00

*Demographic factors*						
*Gender & Age Composition of Household Labor Pool*						
# of Male HH members 15–39	1.14	0.03	1.09	1.20	0.00	14.00
# of Female HH members 15–39	1.36	0.02	1.31	1.41	0.00	8.00
# of Male HH members 40–59	0.47	0.01	0.45	0.49	0.00	2.00
# of Female HH members 40–59	0.50	0.01	0.47	0.53	0.00	5.00
# of Male HH members 60+	0.31	0.01	0.28	0.33	0.00	3.00
# of female HH members 60+	0.20	0.01	0.18	0.22	0.00	3.00
Household head: female, with male†	0.06	0.01	0.05	0.07	0.00	1.00
Household head: female, with no male†	0.02	0.00	0.01	0.03	0.00	1.00
Household is polygamous†	0.23	0.01	0.21	0.25	0.00	1.00
Household size	7.21	0.07	7.07	7.34	2.00	31.00
*Household labor substitutes*						
Agricultural implements and machinery access	0.11	0.08	−0.04	0.26	−0.41	42.27
Total hired labor (Days)	13.20	0.62	11.99	14.41	0.00	210

*Cultural roles*						
*Competing demands on time*						
# of HH members 0–5	1.16	0.03	1.10	1.22	0.00	9.00
# of HH members 6–14	1.91	0.04	1.84	1.98	0.00	10.00
% of female adult HH members suffering from chronic disease	0.09	0.01	0.08	0.10	0.00	1.00
% of male adult HH members suffering from chronic disease	0.02	0.00	0.01	0.02	0.00	1.00
Total land cultivated by the household	0.87	0.02	0.83	0.91	0.01	9.11
% of cultivated land under cereals	0.54	0.01	0.52	0.56	0.00	1.00
% of cultivated land under legumes	0.04	0.00	0.03	0.05	0.00	1.00
% of cultivated land under roots & tubers	0.39	0.01	0.37	0.41	0.00	1.00
% of cultivated land under fruits & vegetables	0.02	0.00	0.02	0.03	0.00	1.00

*Economic reasons*						
Maximum years of schooling among male HH members	7.41	0.12	7.18	7.64	0.00	22.00
Maximum years of schooling among female HH members	5.67	0.11	5.45	5.90	0.00	18.00
Wealth INDEX	−0.90	0.05	−1.00	−0.80	−3.82	7.98
Livestock Ownership (TLUs)	0.03	0.01	0.01	0.04	0.00	12.50
Access to non-farm earned income†	0.67	0.01	0.65	0.69	0.00	1.00
Access to non-farm non-earned income†	0.07	0.01	0.06	0.08	0.00	1.00
% of land owned by female HH members	0.15	0.01	0.13	0.17	0.00	1.00
Travel time to nearest 20 K population center	21.72	0.32	21.08	22.35	0.06	82.78
Household: rural†	0.87	0.01	0.85	0.89	0.00	1.00

**Table B3:** Descriptive Statistics for Niger.

Variable	Mean	Std. Err.	[95% Conf. Interval]		Min	Max
*Outcome variable*						
Household female share of agricultural labor	0.22	0.01	0.20	0.23	0	1

*Survey methodology*						
Female manager in majority of household plots, land weighted	0.07	0.01	0.05	0.08	0	1

*Demographic factors*						
*Gender & Age composition of household labor pool*						
# of male HH members 15–39	0.93	0.02	0.89	0.98	0	7
# of female HH members 15–39	1.14	0.02	1.10	1.19	0	8
# of male HH members 40–59	0.36	0.01	0.34	0.39	0	2
# of female HH members 40–59	0.38	0.02	0.34	0.41	0	5
# of male HH members 60+	0.19	0.01	0.17	0.21	0	1
# of female HH members 60+	0.13	0.01	0.11	0.15	0	3
Household head: female, with male†	0.03	0.00	0.02	0.04	0	1
Household head: female, with no male†	0.04	0.01	0.03	0.05	0	1
Household is polygamous†	0.24	0.01	0.22	0.27	0	1
Household size	6.80	0.09	6.62	6.98	1	29
*Household labor substitutes*						
Agricultural implements and machinery access	1.02	0.04	0.94	1.09	−2	9
Total hired labor (Days)	3.43	0.19	3.06	3.80	0	60
Total exchange labor (Days)	0.45	0.03	0.39	0.51	0	15

*Cultural roles*						
*Competing demands on time*						
# of HH members 0–5	1.86	0.04	1.79	1.94	0	7
# of HH members 6–14	1.86	0.04	1.77	1.95	0	12
% of female adult HH members suffering from chronic disease	0.28	0.01	0.26	0.30	0	1
% of male adult HH members suffering from chronic disease	0.22	0.01	0.20	0.24	0	1
*Household head ethnicity*						
Haoussa†	0.56	0.01	0.53	0.58	0	1
Djerma†	0.20	0.01	0.18	0.22	0	1
Touareg†	0.12	0.01	0.10	0.14	0	1
Total land cultivated by the household	5.33	0.12	5.10	5.56	0.1	38
% of cultivated land under cereals	0.67	0.01	0.65	0.69	0	1
% of cultivated land under legumes	0.29	0.01	0.27	0.31	0	1
% of cultivated land under roots & tubers	0.00	0.00	0.00	0.00	0	0.6
% of cultivated land under fruits & vegetables	0.03	0.00	0.02	0.03	0	1

*Economic reasons*						
Maximum years of schooling among male HH members	2.36	0.08	2.20	2.52	0	21
Maximum years of schooling among female HH members	1.63	0.07	1.50	1.76	0	18
Wealth index	−1.40	0.03	−1.45	−1.35	−3	14
Livestock ownership (TLUs)	0.06	0.01	0.05	0.07	0	6
Access to non-farm earned income†	0.57	0.01	0.55	0.60	0	1
Access to non-farm non-earned income†	0.58	0.01	0.55	0.60	0	1
% of land owned by female HH members	0.09	0.01	0.08	0.10	0	1
Travel time to nearest 20 K population center	1.88	0.03	1.82	1.94	0	8
Household: rural†	0.94	0.00	0.93	0.95	0	1

**Table 1:** Female share of agricultural labor versus total population (%) by country.

Country	Female share of agricultural labor (LSMS-ISA)	Female share of total population	Female Share of agricultural labor (ILO)
Uganda	56	52	54
Tanzania	52	53	54
Malawi	52	51	53
Nigeria	37	50	36
* Northern*	*32*	*49*	
* Southern*	*51*	*51*	
Ethiopia	29	49	–
Niger	24	51	–

Total average⁎	40	51	

Note:

⁎ Population weighted.

**Table 2:** Female share of agricultural labor (%) by crop and country.

Crops	Tanzania	Malawi	Niger	Uganda	Northern Nigeria	Southern Nigeria	Ethiopia	Total⁎
Cereals	52	54	21	55	28	43	28	31
Legumes	54	53	28	59	28	51	23	31
Roots + tubers	52	50		60	50	54		42
Fruits + vegetables + permanent crops	45	49	37	53	36	39	38	34
Non-edible crops⁎	54	47		50	31	61	32	33

Total	52	52	24	56	32	52	29	40

Note: No shares are reported for categories with less than 2% of the observations.

⁎ Population weighted.

**Table 3:** Female share of agricultural labor (%) by activity domain and country.

Activity domain	Tanzania	Malawi	Niger	Uganda	Northern Nigeria	Southern Nigeria	Ethiopia	Total
Land preparation	52	53	18		31	51	26	33
Planting, weeding	53	53	25		31	51	26	33
Harvesting	54	51	28		34	51	37	36

Total	53	52	24	56	32	51	29	40

Note: ^∗^ Population weighted.

**Table 4:** Female share of agricultural labor (%) by age group and country.

Age group	Tanzania	Malawi	Niger	Uganda	Northern Nigeria	Southern Nigeria	Ethiopia	Total⁎
0–15	52	49	25	54	30	46	43	40
15–30	51	54	27	55	32	54	30	40
30–45	56	52	24	56	38	61	30	44
45–60	52	52	23	61	29	50	26	39
60+	51	52	10	54	21	40	15	33

Total	53	52	24	56	32	51	29	40

Note:

⁎ Population weighted.

**Table 5:** Correlates of household female labor share in agriculture in Malawi, Nigeria and Niger.

	Malawi	Nigeria	Nigeria North	Nigeria South	Niger
*Survey methodology*					
Respondent knows (works at least 50% on the plots)	0.04⁎⁎⁎	−0.12⁎⁎⁎	−0.15⁎⁎⁎	−0.07⁎⁎⁎	
	(0.01)	(0.01)	(0.01)	(0.02)	
Respondent female (in at least 50% on the plots)	0.06⁎⁎⁎	−0.02	−0.05	−0.01	
	(0.00)	(0.03)	(0.05)	(0.04)	

*Demographic factors*					
*Gender & age composition of household labor pool*					
# of Male HH members 15–39	−0.06⁎⁎⁎	−0.03⁎⁎⁎	−0.04⁎⁎⁎	−0.01	−0.05⁎⁎⁎
	(0.00)	(0.01)	(0.01)	(0.01)	(0.01)
# of Female HH members 15–39	0.07⁎⁎⁎	0.02⁎⁎⁎	0.02⁎⁎⁎	0.01	0.02⁎⁎⁎
	(0.00)	(0.01)	(0.01)	(0.01)	(0.01)
# of Male HH members 40–59	−0.06⁎⁎⁎	−0.03⁎⁎	−0.03⁎⁎	−0.03	−0.06⁎⁎⁎
	(0.01)	(0.01)	(0.01)	(0.03)	(0.01)
# of Female HH members 40–59	0.09⁎⁎⁎	0.02⁎⁎⁎	0.02⁎⁎	0.03⁎	0.03⁎⁎⁎
	(0.01)	(0.01)	(0.01)	(0.02)	(0.01)
# of Male HH members 60+	−0.06⁎⁎⁎	−0.03⁎⁎	−0.04⁎⁎⁎	−0.02	−0.04⁎⁎
	(0.01)	(0.01)	(0.01)	(0.03)	(0.02)
# of Female HH members 60+	0.10⁎⁎⁎	0.03⁎⁎	0.02⁎	0.04⁎	0.04⁎⁎
	(0.01)	(0.01)	(0.01)	(0.02)	(0.02)
Household head: female, with male†	0.07⁎⁎⁎	0.00	−0.05	0.02	0.10⁎⁎
	(0.01)	(0.04)	(0.07)	(0.05)	(0.04)
Household head: female, with no male†	0.30⁎⁎⁎	0.13⁎⁎⁎	0.11	0.13⁎⁎⁎	0.44⁎⁎⁎
	(0.01)	(0.04)	(0.08)	(0.05)	(0.04)
Household polygamous	0.04⁎⁎⁎	−0.01	−0.01	0.01	0.04⁎⁎⁎
	(0.01)	(0.01)	(0.01)	(0.03)	(0.01)
*Household labor substitutes*					
Agricultural implements and machinery access	−0.00	−0.00⁎⁎	−0.00⁎⁎⁎	−0.00	−0.01⁎⁎
	(0.00)	(0.00)	(0.00)	(0.00)	(0.00)
Total hired labor (Days)	−0.00	0.00⁎	0.00⁎⁎	0.00	−0.00
	(0.00)	(0.00)	(0.00)	(0.00)	(0.00)
Total exchange labor (Days)	0.00				0.01
	(0.00)				(0.01)

*Cultural roles*					
*Competing demands on time*					
# of HH members 0–5	0.01⁎⁎⁎	−0.00	−0.00	−0.01	−0.00
	(0.00)	(0.00)	(0.00)	(0.01)	(0.00)
# of HH members 6–14	0.01⁎⁎⁎	−0.00	−0.00	0.00	−0.00
	(0.00)	(0.00)	(0.00)	(0.01)	(0.00)
% of Female adult HH members suffering from chronic disease	−0.00	0.04⁎	0.05⁎⁎⁎	0.02	−0.01
	(0.01)	(0.02)	(0.02)	(0.04)	(0.01)
% of Male adult HH members suffering from chronic disease	0.00	0.12⁎⁎	0.16⁎	0.09	0.01
	(0.01)	(0.05)	(0.08)	(0.06)	(0.01)
Total land cultivated by the household	−0.00	0.01	0.01	0.01	0.00
	(0.00)	(0.01)	(0.00)	(0.02)	(0.00)
% of cultivated land under cereals	0.03⁎⁎	0.01	0.02	0.01	−0.49⁎⁎⁎
	(0.01)	(0.05)	(0.07)	(0.07)	(0.14)
% of cultivated land under legumes	0.04⁎⁎	0.03	0.04	0.12⁎	−0.47⁎⁎⁎
	(0.02)	(0.05)	(0.07)	(0.07)	(0.14)
% of cultivated land under roots & tubers	−0.00	0.01	0.05	−0.01	−0.80⁎⁎⁎
	(0.04)	(0.05)	(0.07)	(0.07)	(0.17)
% of cultivated land under fruits & vegetables	−0.05	−0.04	−0.03	−0.06	−0.34⁎⁎
	(0.05)	(0.05)	(0.07)	(0.07)	(0.15)

*Household head ethnicity*	Not statistically significant	Not available	Not available	Not available	Statistically significant

*Economic reasons*					
Maximum years of schooling among male HH members	−0.00⁎⁎⁎	−0.00⁎⁎	−0.00	−0.01⁎	−0.00
	(0.00)	(0.00)	(0.00)	(0.00)	(0.00)
Maximum years of schooling among female HH members	0.01⁎⁎⁎	0.01⁎⁎⁎	0.01⁎⁎⁎	0.01⁎⁎	0.01⁎⁎⁎
	(0.00)	(0.00)	(0.00)	(0.00)	(0.00)
Wealth index	0.00⁎	−0.01⁎⁎⁎	−0.01⁎⁎⁎	−0.01	−0.02⁎⁎⁎
	(0.00)	(0.00)	(0.00)	(0.01)	(0.01)
Livestock ownership (TLU’s)	−0.00	−0.03⁎⁎⁎	−0.04⁎⁎⁎		−0.01
	(0.01)	(0.01)	(0.01)		(0.02)
Access to non-farm earned income†	−0.00	−0.02⁎	−0.01	−0.04	−0.01
	(0.00)	(0.01)	(0.01)	(0.03)	(0.01)
Access to non-farm non-earned income†	−0.00	0.02	−0.02	0.05⁎	0.02
	(0.00)	(0.02)	(0.02)	(0.03)	(0.01)
% of land owned by female HH members	0.03⁎⁎⁎	0.33⁎⁎⁎	0.36⁎⁎⁎	0.31⁎⁎⁎	0.13⁎⁎⁎
	(0.01)	(0.04)	(0.07)	(0.05)	(0.04)
Maize production potential (kg/HA, Low Input Maize)	−0.00				
	(0.00)				
Travel time to nearest 20 K population center	0.00	0.00	−0.00	0.00	0.01
	(0.00)	(0.00)	(0.00)	(0.00)	(0.01)
Household: Rural†	−0.02	0.02	0.03	0.01	0.12
	(0.02)	(0.02)	(0.02)	(0.02)	(0.11)
Number of observations	9012	2575	1692	883	2179
R-squared	0.582	0.645	0.604	0.494	0.403
Joint significance of district fixed effects	0.00	0.00	0.00	0.00	0.00

Note:

⁎ p < 0.1.

⁎⁎ p < 0.05.

⁎⁎⁎ p < 0.01.

**Table 6:** Predicted household female labor share in agriculture, controlling for respondent gender and knowledge.

	Malawi	Nigeria
	*Prediction*	*Prediction*
1. Prediction on the whole	56%	32%
2. Respondent knows and respondent female	60%	24%
3. Respondent knows and respondent male	54%	27%
4. Respondent does not know and respondent female	56%	36%
5. Respondent does not know and respondent male	50%	38%
	*Difference*	*Difference*
Total Bias (2 −1):	5%	−7%
Knowledge bias (2–4):	4%	−12%
Gender bias (2–3):	6%	−2%

## Appendix A

See Table A1.

## Appendix B

See Table B1, Table B2, Table B3.

## Footnotes

1 See Backiny-Yetna and McGee, 2015, Kilic et al., 2015, Aguilar et al., 2015, Oseni et al., 2015, Slavchevska, 2015, O’Sullivan et al., 2014.

2 See also the 2003 Maputo Declaration, with the messages reiterated in the 2014 Malabo Declaration on Accelerated Agricultural Growth and Transformation by the African Union.

3 United Nations Economic Commission for Africa (1972, p. 359): “Few persons would argue against the estimate that women are responsible for 60–80 [percent] of the agricultural Labor supplied on the continent of Africa.” A decade later, the Food and Agriculture Organization of the United Nations (FAO) posited that women constitute between 70 and 90% of the agricultural labor force in many Sub-Saharan African countries (FAO, 1984). A later incarnation of the statement surfaced in a 1995 FAO Report: “In Sub-Saharan Africa, agriculture accounts for approximately 21% of the continent’s GDP and women contribute 60–80 [percent] of the Labor used to produce food both for household consumption and for sale.” A related assertion is that women produce 60–80% of the food in developing countries and 50% of the world’s food supply (Momsen, 1991).

4 The LSMS-ISA is a household survey program established with a grant from the Bill and Melinda Gates Foundation to provide financial and technical support to governments in SSA in the design and implementation of multi-topic, national, panel household surveys with a strong focus on agriculture. The program is implemented by the Living Standards Measurement Study (LSMS) in the Development Research Group of the World Bank (www.worldbank.org/lsms).

5 See Bardasi et al. (2011) for an analysis of the effects of proxy versus self-reporting on employment statistics.

6 The female share of agricultural labor for Nigeria overall may seem low at 37% given that the shares for the North and South are 32% and 51% respectively. However, although the population is about evenly split between North and South, a much larger share of the Nigerian population engaged in agriculture resides in the North.

7 All survey rounds, with the exception of Ethiopia Socioeconomic Survey (ESS) 2011/12, are representative at least at the national, rural and urban levels. The ESS 2011/12 round provides representative statistics for rural areas and small towns, which, based on population estimates from the 2007 Population Census, are defined as towns with populations of less than 10,000. See Appendix A for basic information regarding each survey used in the analysis.

8 The Household Questionnaire geo-references the dwelling’s location and collects individual-disaggregated information on demographics, education, health, employment, anthropometrics, and control of off-farm income as well as data on housing, food and non-food consumption and expenditures, and asset ownership, among other topics.

9 Appendix A provides a more detailed, cross-survey overview of the different approaches to the household agricultural labor data collection.

10 In each country, adult household members contributed at least 90% of all labor devoted to crops (including child and hired labor) and 97% of all adult labor. Inclusion of child labor or hired labor does not meaningfully affect the reported labor shares by gender.

11 In all countries, the percentage of missing values is less than 2.5%, except in Nigeria where it reaches 20%. This includes the outliers that have been converted to missing. Additional tests are undertaken to examine the robustness of results from Nigeria.

12 The linear Ordinary Least Squares regression underlying the predictions features the individual-level labor aggregate as the dependent variable, and a range of plot, individual laborer, household and respondent characteristics as independent variables. Plot characteristics include area, agro-ecological potential for maize, primary crop planted on the plot, gender of the manager; input use reflects the use of organic fertilizer, inorganic fertilizer, pesticide, soil quality, agricultural implements index; household characteristics include household size, dependency ratio, head of household’s age, maximum years of schooling in the household; and as worker characteristics their gender, age, age squared are included. The respondent characteristics included are the age, age squared, gender of the respondent and whether the respondent is the manager.

13 See Blair et al. (1991) for a review of the reasons why there may be discrepancies between proxy and self-reports. However, their experiments do not relate to employment issues.

14 Respondents can differ across plots within the household, though typically, there is one respondent per household for all the plots. Information on the respondent is available in Malawi and Nigeria. In Malawi, 47% of the sample households have only male respondents, while 52% have only female respondents. In Nigeria, 79% of the sample households have only male respondents, and 19% have only female respondents.

15 Respondents provide 100% of the agricultural labor on all plots in approximately 12 (Malawi) and 20 (Nigeria) percent of the sample households. Overall, the average share of agricultural labor provided by the respondent is above 50% in 53 and 54% of the households in Malawi and Nigeria respectively. Thus, each respondent provides information on his/her own labor input in the majority of the cases. Robustness of the findings to the use of an alternative measure of the respondent’s knowledgeability, i.e. whether the respondent was the manager in the majority of the household plots, is tested.

16 The household-level database that underlies each estimation is based on the aggregation of individual-level agricultural labor data that are also the basis for estimating the female share of aggregate agricultural labor at the country-level. The findings are robust to the use of the (double-sided censored) Tobit estimator (available upon request).

17 Eq. (1) was also estimated by excluding households with no adult members of the other sex. This did not alter the findings. The results are available upon request.

18 The total land cultivated is based on GPS-based plot area measures, with missing GPS based plot areas imputed following Palacios-Lopez and Yacoubou Djima (2014).

19 Distinguishing between employment category and time use can be key as illustrated by McCullough (2015), who documents how the agricultural labor productivity gap in a number of African countries virtually disappears, when expressing output in per hours worked in a sector as opposed to per person per employment category, suggesting substantial underemployment in the agricultural sector.

20 Adding hired labor does not change the estimated female agricultural labor shares.

21 For Nigeria, where 20% of the observations were missing (including outlier values that were converted to missing), the estimated share remains at 37% even after dropping the outliers. Without any cleaning, the share is estimated at 41% (33% in the North versus 52% in the South).

22 For Ethiopia, the female labor share in agriculture reported in the ILO data base is 41%. This is the only country where the ILO estimate substantially deviates from the LSMS-ISA based estimate (29%). While it cannot be excluded that this is linked to the larger importance of livestock in Ethiopia, the LSMS-ISA data for Ethiopia do not cover major urban centers, and are as such not comparable with the results obtained from ILO. For this reason, they are not included in Table 1. The potential effect of livestock holdings on women’s labor share in crop production is further examined in the multivariate analysis (Table 5). The ILO database does not contain employment/occupation data disaggregated by gender for Niger.

23 Computing female share of agricultural labor by aggregate crop categories, as opposed to individual crops, ensures sufficient number of observations per category on a cross-country basis, but could disguise intra-country variation across crops within an aggregate crop category.

24 The non-edible category includes cactus, coffee, linseed, rape seed, sesame, sunflower, cardamom, ginger in Ethiopia, tobacco, cotton, sunflower, sugar cane in Malawi, cotton in Niger, cotton, gum arabic, rizga, tobacco, jute, oil palm, palm oil, oil bean in Nigeria, cotton and tobacco in Tanzania, sugarcane, cotton, tobacco, coffee, cocoa, tea, ginger, curry, oil palm, and vanilla in Uganda.

25 For Uganda, the reported labor input was not disaggregated by activity domain.

26 Sheahan and Barrett (2014) report that 80% of households in Ethiopia use traction animals and 38% in Niger, compared to less than 20% of households in the other countries.

27 The results are robust to alternative measures of knowledgeability of the respondent, such as whether s/he was the manager in the majority of the household plots. In that case, the predicted female agricultural labor shares range between 61 and 47% in Malawi and between 29 and 37% in Nigeria.

28 The use of modern inputs such as inorganic fertilizer was not found to affect the female labor share.

29 The estimated gender gap in land productivity (defined as the value of agricultural output per unit of cultivated area), based on the LSMS-ISA data, between male and female managed plots (or between male and female individual operators in the case of Ethiopia), stand at 23% for Ethiopia (Aguilar et al., 2015), 25% for Malawi (Kilic et al., 2015), 18% for Niger (Backiny-Yetna and McGee (2015), 4% for northern and 24% for southern Nigeria (Oseni et al., 2015), and 8% for Tanzania (Slavchevska, 2015).

30 According to O’Sullivan et al. (2014), the proportion of plots that are under female management stand at 16% in Ethiopia, 27% in Malawi, 15% in Niger, 10% in Northern Nigeria, 42% in Southern Nigeria, and 21% in Tanzania.

31 This back-of-the-envelope estimate is similar to the estimates provided by Buehren et al. (2015). They project that annual crop output could increase by 2.1% in Tanzania, 2.8% in Uganda, and 7.3% in Malawi as a result of alleviating the gender gap in land productivity.

## References

[b0005] Aguilar A., Carranza E., Goldstein M., Kilic T., Oseni G. (2015). Decomposition of gender differentials in agricultural productivity in Ethiopia. Agric. Econ..

[b0010] Alliance for a Green Revolution in Africa (AGRA) (2012). Investing in Sustainable Agricultural Growth: a Five Year Status Report. http://www.elsevier.com/xml/linking-roles/text/html.

[b0015] Backiny-Yetna, P., McGee, K., 2015. “Gender Differentials in Agricultural Productivity in Niger”. World Bank Policy Research Working Paper No. 7199.

[b0020] Bardasi E., Beegle K., Dillon A., Serneels P. (2011). Do labor statistics depend on how and to whom the questions are asked? Results from a survey experiment in Tanzania. World Bank Econ. Rev..

[b0025] Bezu S., Holden S. (2014). Are rural youth in Ethiopia abandoning agriculture?. World Dev..

[b0030] Blackden, M., Morris-Hughes, E., 1993. “Paradigm postponed: gender and economic adjustment in Sub-Saharan Africa”. Technical Note No. 13, Poverty and Human Resources Division, Technical Department, World Bank Africa Region.

[b0035] Blackden, M., Wodon, Q., 2006. “Gender, time use, and poverty in Sub-Saharan Africa”. World Bank Working Paper No. 73.

[b0040] Blair J., Menon G., Bickart B., Bimer P., Groves R., Lyberg L., Mathiowetz N., Sudman S. (1991). Measurement effects in self vs. proxy responses to survey questions: an information-processing perspective. Measurement Error in Surveys.

[b0045] Buehren, N., Goldstein, M., Kajal G., Kirkwood, D., Slavchevska, V., Smith, D., Torkelsson, A., Westman, M., 015. “The Cost of the Gender Gap in Agricultural Productivity in Malawi, Tanzania, and Uganda”. World Bank Report No. 100234.

[b0050] Dillon, B., Barrett, Christopher B., 2014. “Agricultural Factor Markets in Sub-Saharan Africa: an Updated View with Formal Tests for Market Failure.” World Bank Policy Research Working Paper No. 7117. 10.1016/j.foodpol.2016.09.015 PMC5384445 28413247

[b0055] Doss C., Quisumbing A., Meinzen-Dick R., Raney T., Croppenstedt A., Behrman J., Peterman A. (2014). If women hold up half the sky, how much of the world’s food do they produce?. Gender in Agriculture and Food Security: Closing the Knowledge Gap.

[b0060] Doss, C., Raney, T., Anriquez, G., Croppenstedt, A., Gerosa, S., Lowder, S., Matuscke, I., Skoet, J., 2011. “The Role of Women in Agriculture”. FAO-ESA Working Paper No. 11–02. Retrieved from <http://www.fao.org/docrep/013/am307e/am307e00.pdf>.

[b0065] Food and Agriculture Organization of the United Nations (FAO) (1984). Women in food production and food Security in Africa. Report of the Government Consultation Held in Harare, Zimbabwe, 10-13 July 1984.

[b0070] Food and Agriculture Organization of the United Nations (FAO) (1995). A Synthesis Report of the Africa Region – Women, Agriculture and Rural Development.

[b0075] Food and Agriculture Organization of the United Nations (FAO) (2011). The State of Food and Agriculture 2010–2011 – Women in Agriculture: Closing the Gender Gap for Development.

[b0080] Hertz, T., Winters, P., de la O’Campos, A.P., Quinones, E.J., Azzarri, C., Davis, B., Zezza, A., 2009. “Wage Inequality in International Perspective: Effects of Location, Sector, and Gender.” Paper Presented at the FAO-IFAD-ILO Workshop on Gaps, Trends and Current Research in Gender Dimensions of Agricultural and Rural Employment: Differentiated Pathways out of Poverty. Rome, Italy, 31 March – 2 April 2009. Retrieved from <http://www.fao-ilo.org/fileadmin/user_upload/fao_ilo/pdf/Papers/17_March/Hertz_final.pdf>.

[b0085] Ilahi, N., 2000. “The Intra-Household Allocation of Time and Tasks: What Have we Learnt from the Empirical Literature?” World Bank Policy Research Report on Gender and Development, Working Paper Series No. 13. Retrieved from <http://siteresources.worldbank.org/INTGENDER/Resources/wp13.pdf>.

[b0090] Jackson, C., 2005. “Strengthening Food Policy Through Gender and Intrahousehold Analysis Impact Assessment of IFPRI Multicountry Research.” Impact Assessment Discussion Paper, International Food Policy Research Institute. Retrieved from <http://www.ifpri.org/sites/default/files/publications/ia23.pdf>.

[b0095] Kilic T., Palacios-Lopez A., Goldstein M. (2015). Caught in a productivity trap: a distributional perspective on gender differences in Malawian agriculture. World Dev..

[b0100] Maiga, E., hristiaensen, L., Palacios-Lopez, A., 2015. “Are young people in Africa really leaving agriculture?” Mimeo.

[b0105] McCullough, E., 2015. “Labor Productivity and Employment Gaps in Sub-Saharan Africa”. World Bank Policy Research Working Paper No. 7234. 10.1016/j.foodpol.2016.09.013 PMC5384442 28413252

[b0110] Momsen J.H. (1991). Women and Development in the Third World.

[b0115] Nankhuni F. (2004). Environmental Degradation, Resource Scarcity and Children’s Welfare in Malawi: School Attendance, School Progress, and Children’s Health.

[b0120] Oseni G., Corral P., Goldstein M., Winters P. (2015). Explaining gender differentials in agricultural production in Nigeria. Agric.Econ..

[b0125] O’Sullivan, M., Rao, A., Banerjee, R., Gulati K., Vinez, M., 2014. “Leveling the Field: Improving Opportunities for Women Farmers in Africa.” Retrieved from <http://www-wds.worldbank.org/external/default/WDSContentServer/WDSP/IB/2014/03/14/000333037_20140314131214/Rendered/PDF/860390WP0WB0ON0osure0date0March0180.pdf>.

[b0130] Palacios-Lopez, A., Yacoubou Djima, I., 2014. “Land Areas in the LSMS-ISA Country Datasets: an Application of Multiple Imputation (MI) Methods”. Mimeo.

[b0135] Palacios-Lopez A., Lopez R. (2015). “The gender gap in agricultural productivity: the role of market immperfections”. J. Dev. Stud..

[b0140] Scheffer J. (2002). Dealing with missing data. Res. Lett. Inform. Math. Sci..

[b0145] Sheahan, M., Barrett, C., 2014. “Understanding the Agricultural Input Landscape in Sub-Saharan Africa: Recent Plot, Household, and Community-Level Evidence”. World Bank Policy Research Working Paper 7014.

[b0150] Slavchevska V. (2015). Gender differences in agricultural productivity: the case of Tanzania. Agric. Econ..

[b0155] United Nations Economic Commission for Africa (1972). Human Resources Development Division. “Women: the neglected human resource for African Development”. Can. J. African Stud..

